# The impact of obesity on time spent with the provider and number of medications managed during office-based physician visits using a cross-sectional, national health survey

**DOI:** 10.1186/1471-2458-9-436

**Published:** 2009-11-30

**Authors:** William S Pearson, Kavitha Bhat-Schelbert, Earl S Ford, Ali H Mokdad

**Affiliations:** 1 Division of Adult and Communty Health, Centers for Disease Control and Prevention, Atlanta, GA, USA; 2 Department of Family Medicine, University of Pittsburgh Medical Center, Pittsburgh, PA, USA; 3 Institute for Health Metrics and Evaluation, University of Washington, Seattle, WA, USA

## Abstract

**Background:**

Obesity is associated with morbidity, mortality, and increased health care costs. Few studies have examined the impact of obesity on outpatient office visits. The purpose of this study was to determine if outpatient visits by obese persons required more time with the provider and more prescription medication management compared to visits made by non-obese persons.

**Methods:**

Obesity status was determined for 9,280 patient visits made by persons aged 18 years or older in the 2006 National Ambulatory Medical Care Survey. Multivariate analyses compared obese and non-obese visits, stratified by sex, for duration of the visit and the number of medications mentioned at the visit.

**Results:**

Average duration of visit was higher among visits with patients determined to be obese. However, these differences were not considered significant after statistical testing. Visits made by obese female patients were significantly more likely to involve more than two prescription medications (OR 1.26, 95% CI 1.05 - 1.51) and visits made by obese male patients were significantly more likely to involve more than two prescription medications (OR 1.46, 95% CI 1.16 - 1.83) as compared to visits made by non-obese patients.

**Conclusion:**

Time spent with the provider was found to be greater among visits with obese patients, but not significantly different from visits with non-obese patients. The number of medications for each visit was found to be significantly greater for visits where the patient was considered to be obese. Increased time for the visit and increased numbers of medication for each visit translate into increased costs. These findings document the impact of obesity on our health care system and have great implications on medical care cost and planning.

## Background

Obesity continues to be an important health problem in the United States. Over the past two decades, the trends in the prevalence of overweight and obesity have steadily risen and began to level off in 2002 with 65.7% of adults classified as overweight or obese and 30.2% obese [1]. More recent estimates report that 66.3% of adults are overweight or obese, and 32.2% are obese [2].

With the sustained high rates of overweight and obesity, a heavy cost is levied on the U.S. health care system. According to Finkelstein and colleagues, the direct medical cost of overweight and obesity in the U.S. was $78.5 billion in 1998 [3] and more recent work by Finkelstein and colleagues estimates that medical costs attributable to obesity could run as high as $147 billion in 2008 [4]. Others report that obesity accounts for about 6-10% of medical expenditures in the United States [5-8]. Thorpe and colleagues estimated that between 1987 and 2001, the increase in obesity prevalence accounted for a 12 percent increase in overall health care spending with much of the increase coming from the treatment of co-morbidities of obesity such as hyperlipidemia, heart disease and diabetes [9]. Pearson further noted that as overweight and obesity rates continued to increase from 1995 through 2004, there was an even greater increase in the numbers of visits to office-based physicians for the treatment of diabetes [10]. After controlling for chronic diseases, Raebel estimated that for each unit of BMI increase, costs related to inpatient and outpatient health care increased by 2.3% [11]. These studies indicate that as obesity increases in the U.S. population, more treatment and expense will be rendered for not only obesity itself, but also for its costly co-morbidities.

Many studies have discussed the overall cost impact of obesity, while few have examined specific drivers of cost at the patient-visit level. It is thought that by measuring services at the patient-visit level, costs related to obesity can be quantified. Two measurable costs associated with ambulatory health care include provider time and prescription drug dispensing and management. These two measures were chosen because of their immediate impact on the office visit. Therefore, the purpose of this study was to determine if visits made to office-based physicians by obese persons required more time with the provider and more prescription medications compared to visits made by non-obese persons.

## Methods

### Data

The data for these analyses were taken from the 2006 National Ambulatory Care Medical Survey (NAMCS). The NAMCS is a representative survey of non-federally employed office-based physician visits, including community health centers, conducted yearly by the Centers for Disease Control and Prevention's (CDC) National Center for Health Statistics (NCHS) and is considered to be exempt from review by the CDC's Institutional Review Board. This survey uses a multistage probability design employing the use of geographic primary sampling units (PSUs), physician practices within the PSUs and patient visits within the physician practices. The unit of analysis for this survey is the office visit. Information about the visit including patient demographics, diagnoses, reason for visit, procedures and medications prescribed and monitored during the visit, among other information, are collected by the office staff using patient record forms over a one-week period [12].

The variables that were used in this study included the following: age, race, and sex of the patient, the body mass index (BMI) in kg/m^2 ^(which was calculated by weight and height measurements for the patient at the time of visit), a diagnosis of obesity on the patient's record, payment source, major reason for the visit, total number of chronic conditions at the time of the visit, the number of medications listed for the visit, and the amount of time spent with the provider during the visit.

Age was categorized into four groups including those aged 18-34 years, 35-49 years, 50-64 years, and aged 65 years or older. Race was classified as white, black, and other. Sex was classified as either male or female. Payment source was classified as private pay, Medicare, Medicaid, or other sources such as workers compensation or charity care combined with unknown sources. There were five possible classifications of reason for the visit. They were: a new problem, a routine visit for a chronic problem, a visit made for a flare-up of a chronic problem, a visit made for pre- or post-surgery review, or a visit made for preventive care. The survey questionnaire also determined up to 14 co-morbid chronic conditions for the patient during the visits. They are: arthritis, asthma, cancer, cerebrovascular disease, congestive heart failure, chronic renal failure, chronic obstructive pulmonary disease, depression, diabetes, hyperlipidemia, hypertension, ischemic heart disease, obesity, and osteoporosis.

### Statistical Analyses

All analyses were conducted using SUDANN^® ^to take into account the complex sampling design of the survey [13]. Office visits were stratified into those made by persons with 18.5 kg/m^2 ^≤ BMI <30 kg/m^2 ^and those made by persons with BMI ≥30 kg/m^2 ^or who in lieu of a BMI value had a diagnosis of obesity on the patient record. Visits where the BMI was less than 18.5 kg/m^2 ^or where BMI or obesity status was not able to be determined were excluded from analyses. Estimates were made for all co-variates as well as the average amount of time spent with the provider during the visit and the average number of medications prescribed at each visit. All analyses were stratified by sex of the patient to take into account behavioral differences and care access patterns between males and females.

Time spent with the provider was rounded to the closest five minute interval among those with 18.5 kg/m^2 ^≤ BMI <30 kg/m^2^, which is representative of scheduling practices for office-based physicians. The number of medications mentioned among those with 18.5 kg/m^2 ^≤ BMI < 30 kg/m^2 ^was similarly rounded down to the closest whole number. The proportion of visits that lasted for more than 20 minutes and the proportion of visits where more than 2 medications were prescribed were estimated for both BMI strata and by sex. Differences in these proportions were initially tested using chi-square analyses. These differences were further tested using two adjusted logistic regression models. The first model controlled for age, sex and race of the patient. The second model further controlled for payment source, major reason for the visit, and the total number of co-morbid chronic conditions. All tests were conducted at an α = 0.05.

## Results

A total of 5,806 visits for females and 3,474 visits for males were analyzed. Few differences among the covariates were noted between the two BMI groups. The proportion of visits across the age groups was similar. Black females with BMI > 30 kg/m^2 ^had a greater proportion of visits as compared to Black females with BMI 18.5-29.9 kg/m^2^. Compared to visits with patients with a BMI ≥ 30 kg/m^2^, a greater percentage ofpatient visits with a BMI < 30 kg/m^2 ^were with patients without co-morbid, chronic conditions. Finally, small differences were noted in the two groups based on the average number of medications that were mentioned at the visit and the average amount of time spent with the provider during the visit. Among female visits, those with a BMI ≥ 30 kg/m^2 ^averaged 23.0 minutes in duration compared to 21.4 minutes for those where the BMI was 18.5 - 29.9 kg/m^2^. This represents a 1.6 minute difference. Also among females, visits where the BMI was ≥ 30 kg/m^2^, had on average 3.1 medications mentioned for the visit compared to 2.5 medications mentioned where the BMI was 18.5 -29.9 kg/m^2^. Likewise among male visits, those with BMI ≥ 30 kg/m^2 ^averaged 22.5 minutes in duration compared to 21.2 minutes for visits where the BMI was 18.5 - 29.9 kg/m^2^. This represents a 1.3 minute difference. Finally, among male visits, the average number of medications was 3.1 for visits where BMI ≥ 30 kg/m2 compared to 2.6 where the BMI was 18.5 - 29.9 kg/m2. (Table 1)

**Table 1:** Characteristics of office-based physician visits made by persons with BMI 18.5 - 29.9 kg/m^2 ^and those with BMI ≥ 30 kg.m^2 ^in the United States by sex, 2006

**Body Mass Index**	**18.5-29.9 kg/m^2^**		**≥30 kg/m^2^**	
	**Females**	**Males**	**Females**	**Males**
	n = 3,114	n = 1,985	n = 2,692	n = 1,489
	%, (% S.E.)	%, (%S.E.)	%, (% S.E.)	%, (S.E.)
**Age in Years**				
18-34	20.9, (1.6)	17.5, (1.6)	17.3, (1.3)	10.9, (1.1)
35-49	24.3, (1.2)	23.4, (1.4)	27.6, (1.2)	30.1, (1.7)
50-64	24.9, (1.0)	25.5, (1.4)	32.2, (1.3)	32.7, (1.7)
65+	29.9, (1.8)	33.6, (2.1)	22.9, (1.6)	26.3, (2.1)
**Race**				
White	86.0, (1.3)	84.2, (1.9)	80.6, (2.1)	87.0, (1.6)
Black	9.7, (1.1)	10.2, (1.3)	16.6, (2.1)	8.5, (1.5)
Other	4.2, (0.7)	5.6, (1.6)	2.8, (0.5)	4.5, (1.1)
**Payment Source**				
Private Pay	56.8, (2.0)	47.8, (2.8)	52.8, (2.0)	53.9, (2.6)
Medicare	24.4, (1.8)	27.9, (2.1)	20.8, (1.4)	23.1, (2.1)
Medicaid	7.7, (0.9)	7.0, (1.3)	12.8, (1.3)	7.2, (1.0)
Other/Unknown*	11.2, (1.0)	17.3, (2.4)	13.6, (1.6)	15.9, (2.3)
**Major Reason for Visit**				
New problem	1.1, (0.3)	1.1, (0.3)	1.7, (0.4)	1.0, (0.3)
Chronic problem, routine	34.5, (1.7)	34.9, (2.2)	32.1, (1.6)	35.5, (2.1)
Chronic problem, flare-up	26.4, (1.8)	35.2, (2.2)	33.5, (2.0)	35.8, (2.5)
Pre/post surgery	8.6, (0.8)	8.4, (0.9)	11.3, (1.0)	9.1, (1.1)
Preventive care	6.7, (0.8)	5.8, (1.2)	5.6, (0.8)	5.1, (0.9)
Unknown	22.8, (2.2)	14.5, (2.2)	15.9, (1.5)	13.5, (2.0)
**Number of Co-morbid Chronic Conditions**				
0	40.2, (2.1)	34.3, (2.1)	26.4, (1.7)	24.0, (2.1)
1-2	41.7, (1.7)	44.7, (2.2)	45.4, (1.6)	48.5, (2.1)
3+	18.2, (1.3)	21.0, (1.6)	28.3, (2.0)	27.4, (2.2)
Average number of minutes with the provider**				
	21.4, (0.7)	21.2, (0.7)	23.0, (0.8)	22.5, (0.9)
Average number of medications mentioned at the visit**				
	2.5, (0.1)	2.6, (0.1)	3.1, (0.1)	3.1, (0.2)

Source: 2006 National Ambulatory Medical Care Survey (NAMCS)

S.E. = Standard Error

* Other sources include workers' compensation and charity care

** Mean, (Standard error of the mean)

Chi-square tests comparing the proportion of visits lasting longer than 20 minutes showed no significant differences for either sex. However, for both sexes, significant differences in the number of medications for each visit were higher among those where the patient was considered to be obese. Among female visits where the patient was considered to be obese, 48.5% had more than two prescribed medications compared to 38.6% for those visits where the patient was not considered to be obese (p < .01). Among male visits where the patient was considered to be obese, 50.3% had more than two prescribed medications compared to 40.8% where the patient was not considered to be obese (p < .01). (Table 2)

**Table 2:** Comparisons of estimates for visits with BMI 18.5 - 29.9 kg/m^2 ^and those with BMI ≥ 30 kg/m^2 ^for time spent with the provider during visit and number of medications prescribed during visit by sex, 2006

Females, n = 5806	**18.5 - 29.9 kg/m**^2^	**≥30 kg/m**^2^	
	%, (% S.E.)	%, (% S.E.)	p *
**% of visits where time spent with provider was > 20 minutes**			
	31.7, (2.5)	32.0, (1.8)	.89
**% of visits where number of medications was > 2**			
	38.6, (2.2)	48.5, (2.4)	<.01
Males, n = 3,474	**18.5 - 29.9 kg/m**^2^	**≥30 kg/m**^2^	
	%, (% S.E.)	%, (% S.E.)	p *
**% of visits where time spent with provider was > 20 minutes**			
	27.8, (2.4)	30.9, (2.4)	.16
**% of visits where number of medications was > 2**			
	40.8, (2.5)	50.3, (2.7)	<.01

Source: 2006 National Ambulatory Medical Care Survey (NAMCS)

* Chi-square test α = 0.05

S.E. = Standard Error

Logistic regression models provided significant differences for both female and male visits between the two BMI groups for the likelihood of having more than two prescription medications while controlling for different levels of covariates. No significant differences were seen for amount of time spent with the provider. Among females visits, the first level of models controlling for age and race of the patient demonstrated that visits where the patient was considered to be obese had a 61% greater likelihood of having more than two prescribed medications mentioned at the visit compared to visits where the patient was not considered to be obese (OR 1.61; 95% CI 1.35 - 1.91). Among male visits, the models controlling for age and race demonstrated that visits where the patient was considered to be obese had a 59% greater likelihood of having more than two prescribed medications mentioned at the visit compared to visits where the patient was not considered to be obese (OR 1.59; 95% CI 1.28 - 1.98). (Table 3)

**Table 3:** Adjusted logistic regressions for the likelihood of spending > 20 minutes with the provider during the visit and the likelihood of being prescribed > 2 medications during the visit by sex, 2006

**Females, n = 5,806**		
	**O.R**.	**95% C.I**.
**Amount of time spent with provider during visit > 20 minutes**		
Model 1: controlling for age and race		
BMI ≥ 30 kg/m^2^	0.99	0.83-1.18
BMI 18.5 - 29.9 kg/m^2^	reference	reference
Model 2: controlling for age, race, payment source, number of chronic conditions and major reason for visit		
BMI ≥ 30 kg/m^2^	1.01	0.85-1.20
BMI 18.5 - 29.9 kg/m^2^	reference	reference
**Number of prescribed medications during visit > 2 medications**		
Model 1: controlling for age and race		
BMI ≥ 30 kg/m^2^	1.61	1.35-1.91
BMI 18.5 - 29.9 kg/m^2^	reference	reference
Model 2: controlling for age, race, payment source, number of chronic conditions and major reason for visit		
BMI ≥ 30 kg/m^2^	1.26	1.05-1.51
BMI 18.5 - 29.9 kg/m^2^	reference	reference
**Males, n = 3,474**		
	**O.R**.	**95% C.I**.
**Amount of time spent with provider during visit > 20 minutes**		
Model 1: controlling for age and race		
BMI ≥ 30 kg/m^2^	1.14	0.93-1.40
BMI 18.5 - 29.9 kg/m^2^	reference	reference
Model 2: controlling for age, race, payment source, number of chronic conditions and major reason for visit		
BMI ≥ 30 kg/m^2^	1.13	0.92-1.38
BMI 18.5 - 29.9 kg/m^2^	reference	reference
**Number of prescribed medications during visit > 2 medications**		
Model 1: controlling for age and race		
BMI ≥ 30 kg/m^2^	1.59	1.28-1.98
BMI 18.5 - 29.9 kg/m^2^	reference	reference
Model 2: controlling for age, race, payment source, number of chronic conditions and major reason for visit		
BMI ≥ 30 kg/m^2^	1.46	1.16-1.83
BMI 18.5 - 29.9 kg/m^2^	reference	reference

Source: 2006 National Ambulatory Medical Care Survey (NAMCS)

The second level of models additionally controlled for payment source for the visit, number of co-morbid chronic conditions and major reason for the visit and also showed significant differences between the two BMI groups on the number of medications prescribed. No Significant differences were seen in these models for amount of time spent with the provider. Among females, visits made by patients who were considered to be obese had a 26% greater chance of having more than two prescribed medications mentioned at the visit as compared to visits where the patient was not considered to be obese (OR 1.26; 95% CI 1.05-1.51). Among males in the same model, visits where the patient was considered to be obese had an 46% increased likelihood of having more than 2 prescribed medications mentioned at the visit as compared to visits where the patient was not considered to be obese (OR 1.46; 95% CI 1.16-1.83). (Table 3)

## Discussion

Results from this study have demonstrated the significant impact that obesity has on office-based physician visits. These analyses make a contribution to the literature by demonstrating the impact of obesity at the level of the provider, while many other obesity studies speak to societal costs and overall health system costs of obesity.

The results in this study indicate a greater amount of time spent with the provider during the visit when the patient was obese. On average, for visits where females were obese, the provider spent 1.6 minutes longer with the patient compared to a visit where the patient was not obese. For males, providers spent on average 1.3 minutes longer with obese patients as with non-obese patients. However, these findings for a difference in time were not significant. Although, it appears that medical providers treating obese patients in an outpatient setting prescribed significantly more medications for obese patients compared to non-obese patients, even after controlling for the number of co-morbid conditions and the primary reason for the visit.

Previous research has shown that obesity is related to increases in health care utilization. Two past studies on U.S. cohorts have suggested that the greatest impact of obesity on health care cost and utilization is in outpatient primary care clinical services. The first study, conducted by Andreyeva and colleagues using data from the Health and Retirement Study, found that the average increase in health care costs associated with a BMI of 30 and higher (averaging across all obesity classes) for individuals aged 54-69 years old was 33% [14]. In the second study, Bertakis and colleagues found that (in a prospective study of 509 adult patients) obesity was associated with an increased number of primary care visits, diagnostic services and primary care clinic charges [15]. The same researchers, however, did not find an increase in visit length, but rather that the visit content became more focused on exercise habits and technical tasks rather than disease-specific treatments.

Findings similar to the present study have been demonstrated in other countries using population-based cohorts. von Lengerke and colleagues demonstrated in their study that compared to normal weight persons (18.5 - 24.9 kg/m^2^), those in Obesity Class I (30.0-34.9 kg/m^2^) were more likely to report, over a one-half year period, at least one visit to a general practitioner and that those in Obesity Class II (35.0 - 39.9 kg/m^2^) and those in Obesity Class III (≥ 40 kg/m^2^) had significantly higher odds of having high general practitioner use, defined as eight or more visits in a year [16] Other studies found a positive association between obesity and use of primary health care services [17,18]. In these studies, obese patients required more time with the provider during their visits, and also required a greater number of prescribed medications. Furthermore, these studies found a direct relationship between degree of obesity and amount of health care usage. In simple descriptive analyses, our study showed that visits where the patient was considered to be obese had a longer duration compared to visits where the patient was not considered to be obese. Again though, this difference was not found to be significant. These findings do however point to increased resource utilization.

A second finding of this study was that patients with a BMI ≥ 30 kg/m^2 ^received more prescription medication management during the visit compared to those with BMI < 30 kg/m^2^. In 2007, it was estimated that prescription drugs account for approximately 10 percent of total health expenditures [19] and represent an ever-increasing portion of out-of-pocket expenditures by the individual and an ever increasing proportion of government spending for health care [20].

The analyses in this study are in line with prior research which suggests that prescription medication use is higher in obese persons compared to non-obese persons. For example, a study using the Medicare Current Beneficiary Survey demonstrated that obese Medicare recipients had higher prescription drug costs and higher health care utilization rates compared to non-obese patients [21]. A second example of this phenomenon is the Counterweight Program in the Framingham Heart Study, where Molenaar and colleagues found that not only were obese people using more prescription drugs, but they were more likely to be prescribed medications for hypertension and hyperlipidemia than non-obese patients with the same conditions [22]. Furthermore, a retrospective cohort study examining hospitalizations, outpatient visits and use of other health care services showed that obese individuals were more likely to be hospitalized than non-obese individuals and that their total medical costs were higher due largely to costs from prescription medications [9].

Several limitations should be considered when interpreting the results of this study. The most notable limitation of this study is that exact dollar amount costs were not determined because the dataset does not collect actual costs. However, this study does provide insight into relative resource utilization by persons who are obese, which shows where the costs are being consumed.

Second, the nature of the NAMCS is that the sampling unit is a patient visit, not a person. Therefore, it is possible that one person can be represented in the sample more than once through multiple visits. However, as noted in the survey description, most sampling was conducted in physicians' offices over a limited period of time, specifically a one-week period. Therefore, the likelihood of having one person represented by numerous office visits is low.

A third limitation of this study is that only data on in-person office visits were collected. A significant percentage of medication and disease management is conducted over the telephone, but information on these services was not collected. If this limitation is true, then it is possible that the findings of this study may underestimate the impact of obesity on office-based physician practices.

A fourth limitation of this study was the definition of obesity and the classification of BMI. Some records within this dataset did not contain height and weight. However, they did contain a diagnosis of obesity. Therefore, it was not possible to further stratify persons with obesity into the different obese classes. We were able to provide a distinction between those visits with persons who were obese and those who were not obese. A better detailed measurement of BMI would be useful in determining a more precise effect of increasing increments of BMI on healthcare costs. Even with this limitation, our analyses do provide a rudimentary look at the impact of obesity on outpatient visits.

One future direction for study in this area of health services should include the impact of obesity on other ancillary services such as dietary, physical therapy, and nursing services. Obesity is a health problem that pervades all aspects of health care; not just at the physician-patient nexus. This study and the body of research in this area clearly demonstrate that obesity is a problem that is of great concern for our health care system and will continue to be a problem in the near future. Improvement in lifestyle behavioral interventions and multidisciplinary care management in the outpatient setting may reduce health care costs from obesity and its co-morbid conditions.

## Conclusion

To our knowledge, this is the first nationally representative study using the NAMCS to demonstrate the impact of obesity on provider time and prescriptive practices. The findings in our study and those of others point to a growing problem for individual patients and for our overall health care system due to obesity. Our work used recent ambulatory care data to provide a more focused look at the impact of obesity specifically in the outpatient setting. As our population ages and the demand for the treatment of chronic disease in the outpatient setting grows, healthcare resources will become more constrained. Therefore, it is imperative that obesity, which is a modifiable health issue within the United States, be tackled in order to save on both individual and societal medical costs.

## Competing interests

The authors declare that they have no competing interests.

## Authors' contributions

WSP, KBS, ESF and AHM contributed to the study design and conceptualization, data analysis and interpretation and the writing of the manuscript. All authors have read and approved the final manuscript.

## Pre-publication history

The pre-publication history for this paper can be accessed here:

http://www.biomedcentral.com/1471-2458/9/436/prepub
